# Real-time geometric complexity of language is associated with global functioning across psychosis and major depression

**DOI:** 10.1038/s44277-026-00074-z

**Published:** 2026-11-02

**Authors:** Alexandra Korda, Christina Andreou, Lea Mineur, Frederike Stein, Maia Zhorzholiani, Mihai Avram, Svenja Seuffert, Rieke Roxanne Mülfarth, Thomas Martinetz, Heinz Handels, Iris Sommer, Philipp Homan, Tilo Kircher, Stefan Borgwardt, Wolfram Hinzen

**Affiliations:** 1 Department of Psychiatry and Psychotherapy, University of Lübeck, Lübeck, Germany; 2 Department of Psychiatry and Psychotherapy, Marburg University, UKGM, Marburg, Germany; 3 Institute for Neuro- und Bioinformatics, University of Lübeck, Lübeck, Germany; 4 Institute of Medical Informatics, University of Lübeck, Lübeck, Germany; 5 German Research Center for Artificial Intelligence, Lübeck, Germany; 6 Centers for Clinical Neuroscience and Cognition, University of Groningen, University Medical Center Groningen, Groningen, the Netherlands; 7 Department of Adult Psychiatry and Psychotherapy, Psychiatric University Clinic Zurich and University of Zurich, Zürich, Switzerland; 8 Department of Translation & Language Sciences, Universitat Pompeu Fabra, Carrer Roc Boronat, 138, Barcelona, 08018 Spain; 9 Institut Català de Recerca i Estudis Avançats (ICREA), Barcelona, Spain

**Keywords:** Psychosis, Depression

## Abstract

Language disturbances are prominent across psychiatric disorders, yet their underlying organization and relationship to clinical functioning remain poorly understood. We conceptualized spontaneous speech as a temporally evolving sequence of contextual representations through semantic space, in which successive concepts define transitions between large-language-model embeddings. We characterized the local dynamics of these representations using Lyapunov-inspired measures of sensitivity to perturbation and anisotropy—the extent to which nearby neighboring contextual representations diverge differently depending on direction in representational space. We applied this approach to picture descriptions from 129 German-speaking participants with major depressive disorder (n = 43), schizophrenia spectrum disorders (n = 42), or no psychiatric diagnosis (n = 44). Individual differences in local geometric dynamics predicted multiple clinical dimensions, with the strongest association observed for global functioning, for which the geometric measures explained 51% of the variance. Clinically informative geometric structure was already detectable within the first three sentences of speech, where distinct dynamical profiles were associated with differences in negative and positive symptoms, anxiety, depression, and global functioning. These associations suggest that the dynamics of local representational divergence and contraction during speech reflect broad variation in cognitive and social functioning rather than diagnosis alone. Together, these findings suggest that psychiatric variation is reflected not simply in what people say, but in the dynamics by which semantic representations evolve during ongoing language production. A dynamical-geometric analysis of language may provide a compact, transdiagnostic marker of clinically meaningful variation in psychopathology.

## Lay Summary

This study models speech as trajectories through semantic space using large-language-model embeddings and dynamical systems metrics. In 129 participants with depression, schizophrenia spectrum disorders, or no diagnosis, geometric measures of semantic complexity predicted clinical outcomes, particularly global functioning. Using only the first three sentences, semantic expansion and contraction patterns distinguished symptom profiles. These language-based geometric features explained substantial variance in psychopathology (R² = 0.51), supporting semantic complexity as a transdiagnostic marker of mental health.

## Introduction

Language provides a sensitive behavioural window into brain function, symptoms, diagnosis, and prognosis in mental disorders [1]. Clinical observation and linguistic research show that disruptions in speech at different levels often precede clinical diagnosis and can track illness progression [2]. Speech disruptions are observed at different levels—ranging from parts-of-speech organization to syntactic complexity, semantic organization, and word predictability. These abnormalities are not merely surface features of communication but reflect underlying disturbances in neural circuits supporting prediction, integration, and cognitive control. They capture subtle deviations in language production that often remain undocumented in coarse clinical assessments [3]. Importantly, such metrics can be computed continuously from naturalistic speech, offering a dynamic readout of cognitive and communicative processes as they unfold.

In parallel, machine learning approaches have demonstrated that language-based metrics can be used to classify individuals along the psychosis spectrum with notable accuracy [4] and predict individual symptom scores in the Positive and Negative Symptom Scale (PANSS) with low errors (RMSEs near 1.5 or lower in best-performing models) [5]. Because speech can be collected rapidly, inexpensively, and non-invasively, it offers a powerful prospect for even real-time monitoring—allowing the characterization of patients based on moment-to-moment fluctuations in illness state, cognitive load, or symptom severity. Integrating linguistic analysis with clinical scales has the potential to increase momentary diagnostic precision, reduce clinician burden, and support more personalized, measurement-based dynamic mental healthcare [6–8].

Computational approaches to language in psychiatric disorders have evolved along several complementary lines [9]. Early studies focused on local linguistic and graph-based measures, including semantic coherence and speech graph topology, demonstrating disrupted discourse organization in schizophrenia and associations with symptom severity and functional outcomes [10, 11]. However, these approaches primarily capture local relationships and provide limited information about higher-order semantic structure. Studies used embedding-based methods to represent language in continuous semantic spaces, enabling quantification of global properties such as intrinsic dimensionality and convex hull volume [12–15]. These measures have shown sensitivity to semantic complexity in schizophrenia but summarize language globally rather than capturing its temporal organization. More recent trajectory-based approaches model the temporal organization of speech through semantic space using measures such as semantic drift, path length, and temporal coherence [16, 17]. Although these capture clinically relevant temporal dynamics, they quantify semantic change rather than the local geometric stability of semantic trajectories, leaving fine-grained semantic organization largely unexplored.

Here, we propose a complementary geometric measure of semantic organization, designed to capture how patients’ speech evolves as the interview proceeds over time. Instead of describing the overall (global) geometry or dimensional complexity of the patient’s language, we focus on the local geometric complexity of meaning as it unfolds from one sentence to the next. Embeddings are not treated as isolated observations, but consecutive contextual embeddings are analyzed according to their local geometric organization in semantic space, and the local expansion and contraction patterns using Lyapunov-inspired measures of local dynamics. This representation in the semantic space reflects how an individual’s current context guides subsequent thoughts—providing a window into disorganization or inefficiencies in thought processes that may be clinically relevant. We adapt Lyapunov exponent estimation from dynamical systems theory as a descriptive tool.

Specifically, we use Rosenstein’s algorithm [18] to estimate the Largest Lyapunov Exponent (LLE) and a related parameter (λ) from sliding windows of contextual embeddings derived from speech. In this setting, the LLE indexes the rate at which nearby points in embedding space separate, providing a measure of local sensitivity to small perturbations in semantic representations. The parameter λ indicates the local geometric anisotropy of semantic representations within a given window, which reflects whether semantic representations change similarly in all directions or preferentially along particular directions in the semantic space. By averaging LLE and λ within embedding windows, we obtain window-wise profiles of semantic geometric complexity across speech samples. The specific analysis carried out below takes into consideration the signs of the measures, not the real values. Before clinical translation, it is essential to establish whether such measures are sensitive to clinically relevant variation in psychiatric populations. This initial validation can precede comprehensive normative benchmarking, which will be necessary to support broader clinical interpretation and application.

We test whether such geometric markers of complexity are altered and shared in psychosis and depression groups, and whether they relate to clinical rating scales. Schizophrenia spectrum disorders (SSD) and major depressive disorder (MDD) were selected as prototypical clinical conditions associated with disruptions in language, cognition, and functional outcomes, enabling an initial test of whether dynamical semantic measures capture clinically relevant variation across distinct psychiatric presentations [10]. Although semantic disorganization has been primarily studied in psychotic disorders, growing evidence indicates that MDD also involves alterations in language and cognition. Depression is associated with changes in semantic processing, cognitive flexibility, perseverative thinking, and rumination, which may affect the organization of semantic content during discourse [10, 16, 19].

We hypothesized that symptoms of psychosis and major depression would be associated with differences in geometric complexity and anisotropy of patients’ speech. This hypothesis was motivated by previous work linking alterations in semantic organization and speech dynamics to psychopathology. We additionally explored whether the proposed geometric measures, including λ and LLE, were associated with global functioning and specific symptom dimensions across SSD and MDD. Given that these measures capture fine-grained properties of discourse, we also explored whether their associations with individual clinician-rated symptom dimensions were weaker or less consistent than their associations with broader measures of functioning.

## Materials and methods

### Study participants

We included N = 129 German-speaking participants (aged 18–62) from the FOR2107 MACS cohort (for more details, see Kircher et al. [20], www.for2107.de [20]). Demographic data are presented in Table 1 and the Supplement S1 Section. For the patient groups, inclusion required a lifetime diagnosis of major depressive disorder (MDD) or a schizophrenia spectrum disorder (SSD), including schizophrenia or schizoaffective disorder, as assessed by a semi-structured clinical interview based on the Diagnostic and Statistical Manual of Mental Disorders, Fourth Edition, Text Revision (DSM-IV-TR) [21]. Accordingly, n = 43 participants met criteria for MDD and n = 42 for SSD, no participants met the lifetime criteria for MDD with psychotic features. Healthy control (HC) participants (n = 44) were required to have no lifetime history of, or treatment for, any psychiatric disorder. All procedures were approved by the local Ethics Committee of the Medical Faculty at the University of Marburg (AZ 07/14) according to the Declaration of Helsinki. Written informed consent was obtained from all participants, who received financial compensation for their involvement.

**Table 1:** Descriptive statistics of demographic and clinical variables and non-parametric ANOVA.

	MDD	SSD	HC	total	χ²	df	p
Number of individuals	43	Schizophrenia 22, Schizoaffective disorder 20, SSD total 42	44	129			
Age in years (mean ± SD)	41.88 ± 12.04	40.00 ± 12.26	43.61 ± 12.19	41.86 ± 12.16	3.07	3	0.381
Years of education (mean ± SD)	13.17 ± 3.05	12.13 ± 2.17	13.95 ± 2.99	13.11 ± 2.86	8.12	3	0.044*^2^
Verbal IQ (mean ± SD)	114.66 ± 14.69	113.61 ± 14.59	115.35 ± 13.92	14.53 ± 14.56	1.53	3	0.676
Sex (f/m)	14/29	13/29	19/25	46/83	1.55	3	0.670
Medication Index (mean ± SD)	0.79 ± 1.54	1.90 ± 1.77	0.02 ± 0.15	0.86 ± 1.52	43.7	3	<0.001*^1,2^
Antidepressant medication (mean ± SD)	1.07 ± 2.09	0.79 ± 1.30	0.07 ± 0.46	0.62 ± 1.48	16.7	3	<0.001*^3^
Current chlorpromazine equivalents (mean ± SD)	12.9 ± 60.2	288 ± 363	0.00 ± 0.0	88.1 ± 233	52.9	3	<0.001*^1,2^
SANS (mean ± SD)	10.58 ± 12.04	15.58 ± 14.81	6.28 ± 7.82	11.15 ± 12.62	37.33	3	0.001*^1,2,3^
SAPS (mean ± SD)	3.81 ± 7.76	10.18 ± 12.89	2.21 ± 5.44	5.39 ± 9.77	28.51	3	0.001*^1,2^
HAM-D (mean ± SD)	5.88 ± 7.55	8.15 ± 8.18	2.83 ± 4.22	5.62 ± 7.17	29.56	3	0.001*^2,3^
HAM-A (mean ± SD)	7.93 ± 9.64	9.58 ± 9.19	4.17 ± 6.16	7.19 ± 8.69	30.09	3	0.001*^2,3^
YMRS (mean ± SD)	1.16 ± 3.73	4.95 ± 6.53	0.76 ± 2.28	2.28 ± 4.88	25.46	3	0.001*^1,2^
GAF Score (mean ± SD)	70.63 ± 17.55	56.88 ± 18.93	75.88 ± 14.65	67.99 ± 18.74	51.57	3	**0.001**^**1,2,3**^

*Bonferroni Correction*, a = 0.05 significance level.

^1^SSD vs MDD, ^2^ SSD vs HC, ^3^ MDD vs HC.

### Psychopathological assessment

Table 1 presents the means and standard deviations of the clinical psychopathological assessments. Patients were assessed with psychosis symptom scales, including the Scale for the Assessment of Negative Symptoms (SANS) [22] and the Scale for the Assessment of Positive Symptoms (SAPS), as well as with additional instruments such as the Hamilton Depression Scale (HAM-D [23]), the Hamilton Anxiety Scale (HAM-A) [24], and the Young Mania Rating Scale (YMRS) [25]. To evaluate the level of functionality, global functioning was assessed by trained clinicians using the Global Assessment of Functioning (GAF) scale [26]. Further details on the subscores are presented in the Supplement (Section S2, Table S1).

To assess group differences in clinical symptoms, we conducted a one-way non-parametric ANOVA test followed by pairwise post-hoc comparisons using the Dwass-Steel-Critchlow-Flinger (DSCF) method (Table 1).

### Speech assessment

Spontaneous speech was elicited by the task of describing four pictures from the Thematic Apperception Test (TAT) [27], following a process similar to that described by Liddle et al. [28]. Instead of collecting eight one-minute speech samples, we presented four TAT pictures for three minutes each. This design was chosen to ensure sufficiently long contiguous speech segments within each narrative for the estimation of a temporally evolving sequence of contextual representations through semantic space. Because the present study focuses on local geometric properties of semantic evolution across consecutive utterances, longer within-narrative sequences are required to reliably estimate sliding-window measures (λ and LLE) and to preserve temporal continuity in embedding space [18]. While shorter elicitation schemes (e.g., multiple one-minute samples) increase stimulus diversity, they reduce within-narrative continuity and may limit the stability of trajectory-based estimates (more details in the Supplement, Section S3). Further measures on speech poverty for the analysed dataset are presented in Table S2.

### Speech analysis

#### Language model

**Fig. 1:**
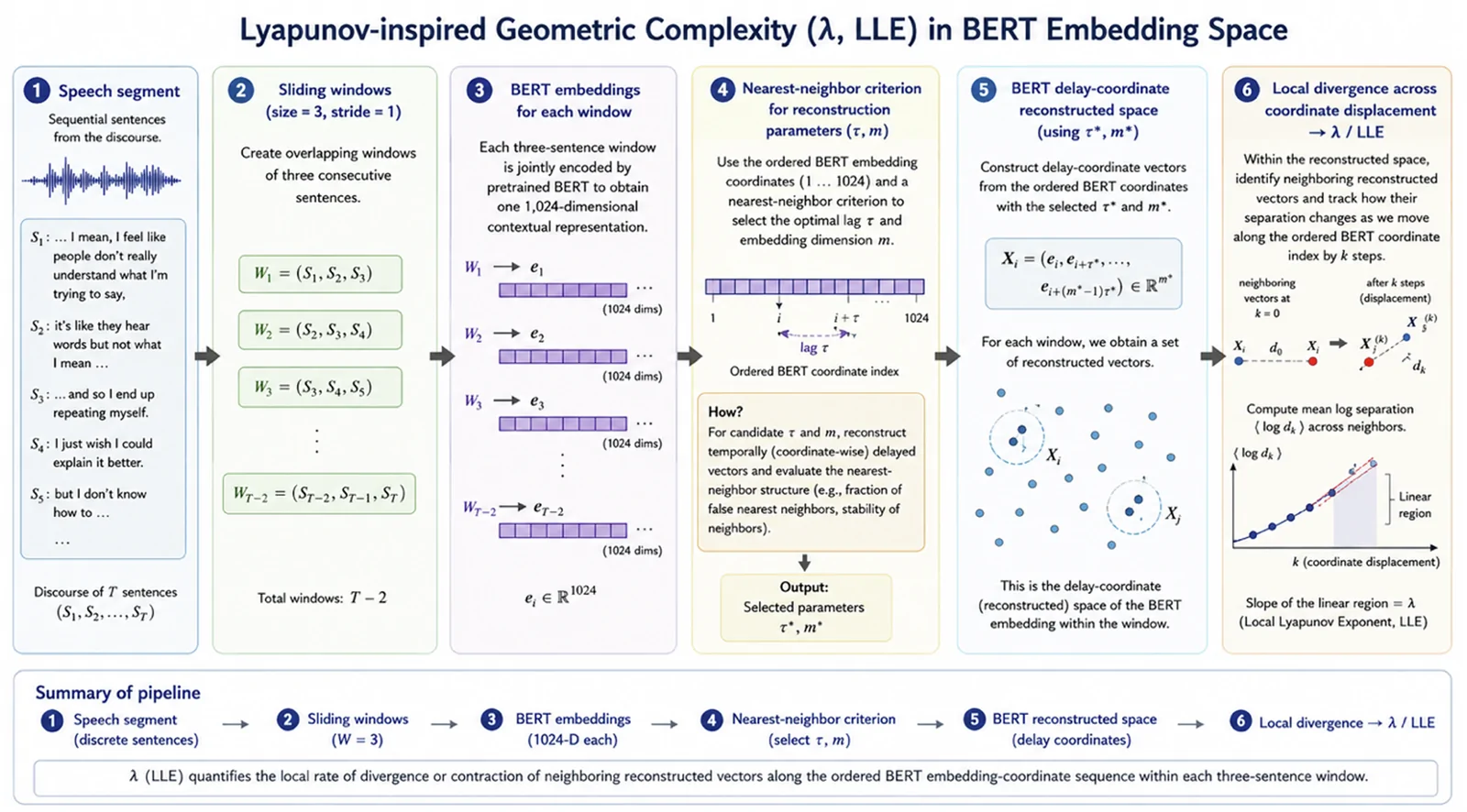
Workflow of the calculation of the geometry of the language complexity across embedding dimensions and discourse windows.

Text embeddings were generated using the pretrained BERT-large-german-cased model GBERT (Hugging Face ID: deepset/gbert-large) [29]. Further details in the Supplement Section S4. Each window in the embedding space is a representation of the sentences in a 1024-dimensional space (see Fig. 1).

#### Geometric language complexity

We used λ and LLE as as Lyapunov-inspired measures of the local geometric complexity measures of contextual representations in embedding space, derived from a pretrained BERT model. These quantities are not interpreted as classical dynamical Lyapunov exponents derived from the temporal evolution of a deterministic system, because the BERT embedding coordinates are not temporal state variables of a known dynamical system. Instead, λ and LLE are interpreted as Lyapunov-inspired measures of local divergence and contraction in the learned representation space.

Formally, we considered the values in the embedding dimensions ${e}_{i},{e}_{j}\in {R}^{1024}$ along the 1024-dimensional vectors. We apply a sliding window over the sequence of sentence embeddings in the discourse. Each window consisted of three consecutive sentences, with a stride of one sentence, resulting in overlapping windows covering the full discourse. Sensitivity analyses were performed using alternative window sizes to ensure robustness of the results. For each three-sentence window, the three sentences were jointly processed using a pretrained BERT model to obtain a single 1024-dimensional contextual representation of that window. Importantly, the analysis involves two distinct levels of ordering. The first ordering is at the within-window level; the 1024 BERT embedding dimensions provide the ordered coordinate indices over which the Rosenstein procedure is applied. These embedding dimensions are not interpreted as temporal observations, and their index does not represent physical time, discourse time, or temporal evolution of a dynamical system. Rather, the ordered embedding-coordinate sequence provides an index along which changes in the separation between neighboring reconstructed vectors can be quantified.

Within each window, the resulting representation was reconstructed into a delay-coordinate representation using an embedding dimension (*m*) and lag (*τ*). Specifically, the ordered coordinate sequence was used to construct delay vectors from embedding-coordinate values separated by lag *τ*. Local similarity was characterized by identifying pairs of reconstructed representations with small Euclidean distance; these pairs were referred to as neighboring reconstructed vectors in BERT embedding space. The parameters *m* and *τ* were selected according to the nearest-neighbor criterion in each analyzed window. Because the reconstruction is performed separately within each three-sentence window, the lag *τ* reflects relative sequence order within the ordered representation coordinates rather than physical or discourse time [18].

We then used the Rosenstein procedure to quantify how the separation between neighboring reconstructed vectors changes along the ordered BERT embedding-coordinate sequence. This was implemented using a Lyapunov-inspired finite-difference formulation applied to the coordinate sequence of the contextual representations.

Within each reconstructed delay-coordinate representation, neighboring reconstructed vectors were identified according to their Euclidean distance. For each neighboring pair, the initial separation was calculated as:

1 $${d}_{i}={{{\rm{||}}}}{e}_{i}-{e}_{j}{{{\rm{||}}}},i\ne j$$

and track how the separation between neighboring reconstructed vectors changes across successive coordinate displacements k along the ordered BERT-coordinate sequence. The separation after a coordinate displacement *k* was then calculated as:

2 $${d}_{i}\left(k\right)={{{\rm{||}}}}{e}_{i+k}-{e}_{j+k}{{{\rm{||}}}},i\ne j$$

where *k* denotes successive displacement along the ordered BERT embedding-coordinate sequence. This formulation is motivated by the logarithmic separation principle used in the Rosenstein approach [18], but is applied here as a geometric finite-difference formulation rather than as a classical dynamical Lyapunov-exponent calculation. The mean logarithmic separation across the identified neighboring pairs was then calculated as:

3 $$\left\langle \log d(k)\right\rangle =\frac{1}{1024}{\sum }_{i=1}^{1024}\log {d}_{i}(k)$$

4 $$\left\langle \log d\right\rangle =\frac{1}{1024}{\sum }_{i=1}^{1024}\log {d}_{i}$$

Following the Rosenstein approach, we estimated the local rate of change of logarithmic separation as a function of displacement along the ordered BERT embedding-coordinate sequence:

5 $$d\left(k\right)=d{e}^{\lambda k}$$

The local geometric divergence parameterλ was estimated as the slope of the linear region of the relationship between mean logarithmic separation and coordinate displacement:

6 $$\left\langle \log d(k)\right\rangle =\left\langle \log d\right\rangle +\lambda k$$

Thus, λ quantifies the local rate of divergence or contraction of neighboring reconstructed vectors within a given three-sentence speech window, evaluated along the ordered BERT embedding-coordinate sequence. Positive values indicate increasing separation, whereas negative values indicate decreasing separation over the evaluated coordinate range. The Rosenstein procedure therefore yields one λ estimate for each speech window, rather than a separate temporal λ trajectory across the 1024 embedding dimensions. For each three-sentence window, the resulting λ estimate was used to characterize the local geometric divergence or contraction of contextual representations within that window:

7 $${LLE}=\frac{\varDelta \left(\left\langle {logd}\left(k\right)\right\rangle \right)}{\varDelta k}$$

Here, LLE denotes the resulting window-level Lyapunov-inspired geometric index, summarizing the local rate of divergence or contraction among similar contextual representations within the speech segment. The sequence of these estimates across the discourse is ${\lambda }_{1},{\lambda }_{2},\ldots ,{\lambda }_{n}$, where *n* is the number of the three-sentence speech windows. This constitutes the second level of ordering: the between-window ordering, in which the window-level estimates are sequentially ordered according to their position in the original discourse. At this between-window level, the windows are temporally ordered because they are generated sequentially from the discourse using a sliding window with a stride of one sentence. Consequently, the resulting window-level λ values form a temporally ordered sequence of geometric indices. Temporal comparisons and changes in λ are therefore made between successive speech windows, and not across the 1024 BERT embedding dimensions. The Lyapunov-inspired measures quantify how neighboring embeddings diverge or converge locally within this embedding space, reflecting the degree to which small perturbations in linguistic context are associated with divergence or contraction of naturally occurring neighboring contextual representations along the ordered embedding-coordinate sequence. In this context, “perturbation” refers to small changes in linguistic content between contextual representations in the embedding space associated with nearby linguistic content within a window. These changes appear as neighboring points in the contextual embedding space and are therefore treated as local representational perturbations. This terminology does not imply a controlled perturbation of a dynamical system; rather, it refers to naturally occurring local variation in linguistic context and its corresponding geometric representation.

In this framework, increased local divergence may reflect reduced contextual constraint in speech production, while increased local contraction may reflect repetitive or overly constrained semantic organization. Both patterns have been implicated in thought disorder, rumination, and reduced cognitive flexibility in psychiatric conditions (Figures S1 and S2 in the Supplement Section S4).

### Statistical analysis

#### Elastic net regression model

We investigated the *signs* of λ and LLE from each embedding window as the geometric indices of local semantic organization to predict clinical symptom severity. Due to differences in speech duration, the number of windows in each patient was different. We therefore computed a first-order Markov transition matrix describing the probability of transitioning between sign states across consecutive windows for λ and LLE sequences. Transition counts were accumulated across the sequence and subsequently row-normalized such that each row represented conditional transition probabilities given the current state:

$${T}_{{ij}}=\frac{{{\mbox{Number of transitions from state}}} \, i \, {{\mbox{to}}} \, j}{{{\mbox{Total transitions from state}}} \, i}$$

where state *i* corresponds to a positive sign and state *j* to a negative sign. This normalization ensures that each row sums to 1 and reflects the probability of remaining in or switching from a given state. Thus, the number of features for each subject was the same.

To assess the internal validity and generalizability of the mathematical measures, we conducted a leave-one-out cross-validation (LOOCV), where an elastic-net regression model was trained with combined predictors and then tested for each held-out observation (details in the Supplement Section S5).

We controlled for diagnosis, age, sex, years of education, IQ, and medication. Although there were no statistically significant differences in word count, sentence count, and window counts between groups, we used these three measures as further covariates in the model. Demographic covariates were included directly as predictors to avoid information leakage. All predictions were obtained using nested cross-validation. We further explored the predictive power of the transition states of signs of λ and LLE values, by extracting them also from the *inverse* sequence of the dimensions in each window and subject. The aim was to investigate the effect of the sequence of the embedding windows and dimensions in the model.

To improve interpretability of the continuous measures λ and LLE, we additionally considered their sign structure as a coarse-grained representation of local geometric regime. This yields a 2×2 state space defined by whether each window exhibits positive or negative λ (local divergence vs contraction) and positive or negative LLE (average divergence rate vs contraction). This discretization allows us to characterize whether subjects predominantly occupy consistent or mixed geometric regimes across discourse.

#### Clustering analysis

Additionally, we investigated the statistical significance of the discriminative power of λ and LLE with regard to the patient’s psychopathology using their signs of each window, both separately and combined. We define a joint sign state space based on λ ∈ {+, −} and LLE ∈ {+, −}, resulting in four possible geometric regimes of local semantic organization. The resulting four combinations correspond to distinct regimes of semantic organization: (+ /+) globally and locally expanding structure, (− /−) globally and locally constricted structure, and mixed regimes (+/− and −/+), reflecting dissociation between local and aggregate geometric dynamics. Clustering is therefore used to group subjects based on their dominant occupancy patterns across these four geometric regimes. Each cluster represents a distinct distribution of occupancy across the four sign-based regimes, rather than a single discrete state.

Specifically, we ran three clustering approaches based on the windows analysed, using: Case A) Signs across all windows, using zero imputation for the subjects with different window lengths; Case B) Signs of the first embedding windows, and Case C) Signs of the last embedding window, for each speech sample. We included an exploratory analysis of positional effects in discourse structure, given evidence that window-initial and window-final language can differ systematically in coherence and elaboration. We then calculated the Silhouette coefficient to evaluate how well each subject fitted within its assigned cluster based on their clinical scores, considering both the cohesion (how close the point is to other points in the same cluster) and the separation (how far the point is from points in the nearest neighbouring cluster). Cluster validity was assessed using permutation tests on mean Silhouette scores and between-to-within dispersion ratios, comparing observed clustering solutions against null distributions obtained by label randomization. We applied 1000 permutations to test the statistical significance of the clusters.

## Results

### Elastic-net regression analysis

Elastic-net regression models using the transition states of signs of λ and LLE from each window significantly predicted all clinical symptom scores, as demonstrated by permutation testing with FDR_correction (all p < 0.01). Explained variance (R²) ranged from modest to substantial across scales, with the strongest predictive performance observed for global functioning (GAF; R² = 0.51), followed by negative symptoms (SANS; R² = 0.32), affective and anxiety symptoms (HAM-D, HAM-A; R² ≈ 0.09–0.19), positive symptoms (SAPS; R² = 0.13), and manic symptoms (YMRS; R² = 0.07) (Table 2). We also tested the subscores of the clinical scores, as well as positive and negative formal thought disorders, without reaching statistically significant predictions (Supplement Table S2). When using the transition states of the inverse sequence of the dimensions, model predictions were significant for all scores and especially for the global functionality (GAF; R² = 0.49), thus showing a similar prediction accuracy. This means that the direction of the dimensions imported into the Lyapunov equation doesn’t affect the prediction scores. We proceed using the original sequence of dimensions, which provided the highest explained variance.

**Table 2:** A) Results from the elastic-net regression analysis predicting clinical scores from transition states of signs of λ and LLE of each window. B) Results from the elastic-net regression analysis predicting clinical scores from transition states of signs of λ and LLE of each window using the inverse sequence of dimensions.

A			
Clinical scores	R^2^	Permuted_FDR *p < 0.01	RMSE CI
SANS	**0.32**	<0.00*	10.20 [8.17–12.10]
SAPS	0.19	<0.00*	8.13 [5.80–10.44]
YMRS	0.06	<0.00*	3.91 [2.78–5.15]
GAF	**0.51**	<0.00*	12.37 [10.92–14.45]
HAM-A	0.09	<0.00*	8.32 [6.70–9.88]
HAM-D	0.09	<0.00*	6.16 [4.89–7.44]

B			
Clinical scores	R^2^	Permuted_FDR *p < 0.01	RMSE CI
SANS	**0.31**	<0.00*	11.55 [9.24–13.83]
SAPS	0.10	<0.00*	8.47 [5.97–10.74]
YMRS	0.06	<0.00*	3.88 [2.82–4.96]
GAF	**0.49**	<0.00*	13.42 [11.94–14.99]
HAM-A	0.13	<0.00*	8.93 [7.25–10.67]
HAM-D	0.19	<0.00*	6.81 [5.28–8.35]

### Clustering analysis

**Case A**: For clustering the subjects using the signs of λ and LLE, separately, permutation testing of Silhouette scores indicated that the clusters with negative λ and the clusters with positive λ, independent of the signs of LLE, were significantly stronger than expected under random labelling (p = 0.01) for several windows. To have meaningful interpretations, comparable windows need to be considered. Thus, we applied the same approach for the first window (Case B) and the last window of speech (Case C).

**Case B**: Using the first embedding window only, the patient’s profiles based on the clinical scores were differentiated using the sign of λ. There were no statistically significant differences using the sign of the LLE or combining the signs of both measures.

**Case C**: Using the last embedding window only did not reach statistically significant discrimination of the clusters. Clustering using the LLE values, whether separately or combined with λ, did not yield significant results in all three cases either.

Descriptive results of Case B using the first window and the results from the non-parametric ANOVA test between the two clusters are presented in Tables 3 and 4.

**Table 3:** Mean value and 95% confidence intervals of the clinical symptoms using the sign of λ in the first window.

Cluster	SANS	SAPS	HAM-D	HAM-A	YMRS	GAF
Positive λ	17.5 [11.9–23.0]	7.12 [2.80–11.4]	8.15 [4.41–11.9]	10.1 [5.65–14.6]	3.19 [0.86–5.53]	56.8 [49.7–63.9]
Negative λ	8.55 [5.83–11.3]	4.29 [2.36–6.21]	5.09 [3.71–6.48]	6.67 [4.79–8.55]	1.42 [0.67–2.18]	70.1 [66.0–74.2]

**Table 4:** Results of non-parametric ANOVA (Kruskal-Wallis) test between the two clusters using the sign of λ in the first window.

	χ²	df	p
Age	0.00	1	0.976
Sex	0.035	1	0.852
Years of education	3.67	1	0.055
SANS	11.71	1	< 0.001*
IQ	2.32	1	0.128
GAF	9.81	1	0.002*
YMRS	1.55	1	0.213
HAM-A	1.91	1	0.167
SAPS	3.29	1	0.069
HAM-D	2.01	1	0.156
Window Count	0.08	1	0.771
Sentence Count	0.08	1	0.771
Word count	0.00	1	0.986
Medication Index	13.98	1	<0.001*
Antidepressant medication	9.22	1	0.002*
Current chlorpromazine equivalents	9.20	1	0.002*

*Bonferroni Correction, Level of significance a = 0.05.

Using this clustering approach, statistically significant differences were identified in SANS and GAF between the two clusters. A positive (negative) sign of λ in the first window indicates high (low) SANS and low (high) GAF.

We further explored Case B using the combination of the signs of λ and LLE. Specifically, we designed four exploratory groups according to the sign of the λ in the first window and the sign of the maximum value of the LLE. Group 1: Positive λ-Positive LLE, Group 2: Positive λ-Negative LLE, Group 3: Negative λ-Positive LLE, Group 4: Negative λ-Negative LLE. In Table 5, the mean values of the clinical scores and the 95% confidence intervals for each group, assuming sample means follow a t-distribution, are presented. In Table 6, the results from the non-parametric ANOVA test between four groups are presented. The analysis identified group-level differences in education, SANS, GAF, HAM-A, SAPS, and HAM-D. However, these findings should be considered exploratory, particularly because the group sizes were highly unequal.

**Table 5:** Mean value and 95% confidence intervals of the clinical symptoms.

Group	SANS	SAPS	HAM-D	HAM-A	YMRS	GAF
1	20.4 [17.4–23.4]	7.80 [−2.45–18.1]	11.6 [0.132–23.1]	13.8 [4.79–22.8]	3.00 [−3.33–9.33]	50.2 [35.5–64.9]
2	16.8 [9.86–23.7]	6.95 [1.79–12.1]	7.33 [3.09–11.6]	9.24 [3.89–14.6]	3.24 [0.485–5.99]	58.4 [50.0–66.8]
3	7.97 [4.15–11.8]	4.06 [1.79–11.6]	4.65 [2.48–6.81]	6.26 [3.28–9.24]	1.61 [0.458–2.77]	69.7 [63.3–76.2]
4	9.06 [5.05–13.1]	4.49 [1.34–7.63]	5.49 [3.62–7.35]	7.03 [4.52–9.54]	1.26 [0.22–2.30]	70.4 [64.9–76.0]

**Table 6:** Results of the non-parametric ANOVA test between four groups formed by possible combinations of positive and negative lambda and LLE values.

	χ²	df	p
Age	4.28	3	0.232
Sex	4.68	3	0.196
Years of education	10.92	3	0.012*
SANS	17.36	3	< 0.001*
IQ	4.28	3	0.232
GAF	15.18	3	0.002*
YMRS	4.97	3	0.174
HAM-A	8.64	3	0.034*
SAPS	8.79	3	0.032*
HAM-D	8.90	3	0.031*
Window Count	12.21	3	0.007*
Sentence Count	12.21	3	0.007*
Word count	5.12	3	0.163
Medication Index	15.75	3	0.001*
Antidepressant medication	9.65	3	0.022*
Current chlorpromazine equivalents	10.67	3	0.014*

^*^Bonferroni Correction, Level of significance a = 0.05.

More detailed inspection showed that in Group 1, subjects present SANS > 18, suggesting notable negative-symptom impairment. However, only 4 subjects were included in this group, substantially limiting the reliability and interpretability of this pattern. Accordingly, the four-group analysis should be regarded as hypothesis-generating rather than as evidence of distinct or clinically established psychopathological profiles. In turn, Group 3 was associated with low or subthreshold positive symptoms (SAPS < 25). Moreover, independently of the λ values, if the LLE presents a positive value (as in Group 1 and Group 3), then 52 subjects out of 55 in these groups present SAPS < 25. Additional results with the subscores are presented in Supplementary Tables S3-S4.

## Discussion

Inspired by dynamical systems and chaos theory, this study has introduced and clinically evaluated a framework for quantifying geometric language complexity in psychiatric speech. Rather than developing a diagnostic tool, we examined whether embedding-derived semantic trajectories capture clinically relevant variation in symptoms and functioning across SSD and MDD. Our findings extend existing computational approaches by incorporating the sequential organization of discourse into the analysis of contextual representations. Although the measures demonstrated promising associations with clinically relevant features, further validation is needed in larger and more diverse samples, including assessment of demographic influences and test–retest reliability. Unlike approaches relying on global linguistic complexity or semantic coherence, we characterized local semantic complexity and anisotropy using trajectory-based representations from contextual language embeddings. Rather than representing invariant dynamical parameters, Lyapunov exponents were treated as local descriptors of complexity along induced semantic trajectories. Sequential embedding analysis captures changes in topic, syntax, semantic coherence, and representational structure, enabling characterization of both local and global geometric properties of discourse.

The proposed complexity measures predicted clinical symptom dimensions across MDD, SSD, and HC, highlighting language-based markers of psychopathology independent of diagnosis. Predictive performance was strongest for global functioning, suggesting that language complexity is particularly sensitive to overall functional impairment. Consistent with previous work [30–33], we found that different symptom dimensions were associated with distinct linguistic features: negative symptoms with impoverished lexical, syntactic, and discourse organization [34–36], whereas associations with positive symptoms were weaker and more context-dependent. Global Assessment of Functioning (GAF) is a broad clinician-rated measure of overall psychosocial functioning and does not directly index linguistic or cognitive processes. GAF should thus be interpreted as a coarse outcome measure rather than a specific proxy of cognitive or linguistic processes. Associations with GAF provide preliminary evidence that the proposed linguistic complexity measures are related to overall functional status. Given that GAF is a broad, symptom-conflated measure that does not directly assess linguistic or cognitive processes, these associations should not be interpreted as evidence for specific underlying cognitive or linguistic mechanisms. Moderate prediction of negative, affective, and anxiety symptoms indicates that these symptom dimensions are well captured by the linguistic features. In contrast, lower explained variance for positive and manic symptoms may reflect the more episodic, context-dependent, and clinically heterogeneous nature of these symptom domains, which may not be reflected in a single short speech task. Of note, due to the extensive protocol of this study, neither acute nor highly psychotic patients were included, limiting the representativeness of symptom profiles of this study. Higher levels of functional impairment are likely accompanied by reductions in semantic richness, structural organization, and adaptability of language, consistent with broader cognitive and social difficulties that impact everyday functioning. These findings align with the view that language may function as a proxy reflecting multiple underlying cognitive and affective processes that extend beyond symptom-specific ratings [37].

The lack of a robust association between the proposed semantic organization metric and formal thought disorder (FTD) ratings may reflect differences in construct granularity. FTD ratings (Table S1) are based on global clinical impressions and may not capture subtle or temporally localized disruptions in semantic organization. In contrast, the proposed metric provides a continuous representation of semantic structure, suggesting that clinically rated FTD and embedding-derived geometric organization capture related but distinct aspects of language. The identified clusters provide additional granularity, illustrating how variations in lexical diversity, syntactic structure, and semantic coherence correspond to symptom severity across these domains. Statistically significant differences were observed with negative symptoms for psychosis, depressive symptoms and anxiety, as well as, positive symptoms for psychosis and global scores between clusters.

Using only the first speech window, the proposed metrics showed associations with symptom severity and functioning (negative λ indicating lower severity and higher functioning), suggesting that clinically relevant information may be detectable early in the discourse. As shown in Table 6, the exploratory four-group comparison yielded differences in SANS, GAF, HAM-A, SAPS, and HAM-D scores. The concentration of discriminative information in the first window indicates that the proposed measures may be particularly sensitive to early differences in contextual representation. However, the mechanisms underlying this pattern remain unclear, and the present findings do not allow us to determine whether it reflects differences in planning, semantic organization, or other aspects of speech production.

### Limitations

These exploratory measures require further validation before their potential utility for longitudinal monitoring or risk stratification can be established. Causality cannot be inferred, and the findings require replication. In the MDD group, participants were recruited based on lifetime diagnosis, with generally mild depressive symptoms at assessment (HAM-D = 5.88 ± 7.55), indicating that the observed patterns may reflect both trait- and state-related effects. Longitudinal studies are needed to determine whether these measures track symptom fluctuations. Diagnostic heterogeneity, including the remitted status of many MDD participants and potential overlap with schizophrenia-spectrum disorders, should also be addressed in larger, more homogeneous cohorts. Verbal IQ may have overestimated cognitive ability, limiting generalizability to more severely impaired populations. The proposed measures also require psychometric validation in large normative samples, including assessments of test–retest reliability, demographic effects, and cross-linguistic generalizability. Although age was included as a covariate, nonlinear developmental effects could not be examined. Given the exploratory nature of this study, no a priori power calculation was performed. Larger, more diverse cohorts and assessment of potential confounders, including substance use, are needed to establish the robustness and generalizability of these findings.

### Conclusions

Geometric complexity measures derived from embedding trajectories may provide sensitive markers of psychopathology by capturing local fluctuations in semantic stability. Although this study focused on group differences and symptom associations, these measures may have potential for longitudinal monitoring as symptoms change. Further validation is needed to determine their predictive value and clinical utility. Translation into clinical practice will require validation in longitudinal samples, the establishment of clinically meaningful thresholds, and assessment of potential confounding factors, including medication status and comorbidities.

## Supplementary information

Supplementary Material

## Data Availability

Individual participant data that underlie the findings of this study are available upon reasonable request from the corresponding author. The data are not publicly available due to their containing of information that could compromise the privacy of study participants.
