# Supplementary material for: Real-time geometric complexity of language is associated with global functioning across psychosis and major depression
Source: NPP Digit Psychiatry Neurosci. 2026 Nov 2;4:22. doi: 10.1038/s44277-026-00074-z (PMC13728208; doi:10.1038/s44277-026-00074-z)
Supplement: Supplementary file 1 [file 44277_2026_74_MOESM1_ESM.docx]

**Supplementary Material**

**S1. Study Participants**

Participants were recruited from inpatient and outpatient units of the university hospital in Marburg, from departments of collaborating local hospitals within a 50 km radius, and through advertisements in local newspapers and flyers. General inclusion criteria were an age between 18 and 65 years, sufficient German language proficiency, and the ability to provide written informed consent. Exclusion criteria comprised a verbal IQ < 80, a history of head trauma or loss of consciousness, severe medical conditions (including cancer, autoimmune diseases, or infections), neurological disorders, current substance dependence, and current benzodiazepine use [11]. No additional inclusion criteria regarding illness phase or duration of illness were applied. Participants with MDD were included based on a lifetime diagnosis. Current depressive symptom severity at the time of speech acquisition was assessed using the Hamilton Depression Scale (HAM-D [12]). The mean HAM-D score in the MDD group was 5.88 ± 7.55, indicating generally mild to moderate levels of depressive symptoms at the time of assessment (34 MDD currently in remission based on the DSM-IV-TR).

**S2. Psychopathological Assessments**

All assessments were conducted by trained clinical raters. Interrater reliability was evaluated for all clinician-rated scales listed above and quantified using intraclass correlation coefficients (ICC). Reliability was assessed based on independent ratings by five raters on a subset of participants and showed consistently good agreement, with ICC values > 0.86 for all scales. HC had more years of education than the SDD group, while in the scales for typical symptoms for schizophrenic disorders (SANS and SAPS), SSD showed significantly higher scores than HC. In comparison to HC, SDD also showed significantly higher scores in HAM-D, HAM-A, and YMRS. Regarding the GAF, SSD had significantly lower scores than HC and MDD.

**Table S1**: Descriptive statistics of demographic and clinical variables and non-parametric ANOVA.

|  | MDD | SSD | HC | total | χ² | | | df | | | p | | | |  |
| --- | --- | --- | --- | --- | --- | --- | --- | --- | --- | --- | --- | --- | --- | --- | --- |
| Number of individuals | 43 | Schizophrenia 22  Schizoaffective disorder 20  SSD total 42 | 44 | 129 | |  | | |  | | | |  |  |  |
| SANS_Negative_FTD  (mean ± SD) | 1.63  ± 2.34 | 2.80  ± 2.03 | 0.33± 0.82 | 1.67  ± 2.49 | | | 6.86 | | | 3 | **0.001*^2^** | | | | |
| SANS_ Affective flattening  mean ± SD) | 1.68  ± 2.24 | 2.83  ± 3.29 | 0.48  ± 1.34 | 10.59  ±2.45 | | | 10.78 | | | 3 | **<0.001*^2^** | | |  |  |
| SANS_Alogia  mean ± SD) | 1.41 ±1.91 | 2.52  ± 2.65 | 0.33  ± 0.81 | 1.48  ± 2.14 | | | 14.51 | | | 3 | | **<0.001*^3^** | |  |  |
| SANS_Apathy  mean ± SD) | 2.13  ±2.55 | 3.65  ±2.78 | 0.13  ±0.45 | 2.03  ±2.61 | | | 31.10 | | | 3 | | **<0.001*^1,2,3^** | |  |  |
| SANS_Anhedonia  (mean ± SD) | 1.30 ±2.42 | 2.64  ±2.55 | 0.13  ±0.45 | 1.37  ±2.29 | | | 17.75 | | | 3 | | 0.048 | |  |  |
| SANS_Attention_Deficit  (mean ± SD) | 1.50 ±1.96 | 0.80  ±1.37 | 0.54  ± 1.06 | 1.02  ±1.62 | | | 5.81 | | | 3 | | **0.002*^3^** | |  |  |
| SANS_withoutAttention_Deficit  (mean ± SD) | 8.39  ±8.39 | 19.0  ±13.6 | 1.46  ± 3.06 | 10.0  ±11.7 | | | 12.6 | | | 3 | | **<0.001*^1,2,3^** | |  |  |
| SAPS_Positive_FTD  (mean ± SD) | 2.08  ± 3.53 | 5.43  ± 6.60 | 0.79  ± 1.64 | 2.84  ± 4.81 | | | 1.20 | | | 3 | | **0.001*^1,2^** | |  |  |
| SAPS_Hallucinations | 0.053  ± 0.23 | 2.187  ± 4.67 | 0.00  ± 0.00 | 0.728  ± 2.82 | | | 19.6 | | | 3 | | **0.002*^1,2^** | |  |  |
| SAPS_Delusions | 0.078  ± 0.36 | 2.38  ± 2.83 | 0.00  ± 0.00 | 0.739  ± 1.87 | | | 29.6 | | | 3 | | **<0.001*^1,2^** | |  |  |
| SAPS_Bizarre  Behavior | 0.158  ± 0.55 | 0.567  ± 0.897 | 0.00  ± 0.00 | 0.250  ± 0.657 | | | 13.6 | | | 3 | | **0.006*^1,2^** | |  |  |

*Bonferroni Correction,* a=0.05 significance level

^1^ *SSD vs MDD*, ^2^ *SSD vs HC*, ^3^ *MDD vs HC*

**S3. Speech Assessment**

Participants were instructed to describe what might be happening in each picture. A one-minute break was provided between images, during which the instructions were repeated before the next picture was shown. If participants paused before the three minutes had elapsed, the instructor used non-directive prompts (e.g., “How do people feel?”, “What could happen next?”). All speech samples were audio-recorded using an Olympus WS-853 and transcribed with f4transkript (<https://www.audiotranskription.de/f4transkript/>). The transcribers were blinded to participants’ diagnoses.

**Table S2:** Average values and standard deviation of the window, sentence, and word counts from the speech of all patients in each group. Statistically significant differences between groups are identified using one-way ANOVA and Kruskal-Wallis tests.

|  | MDD | SSD | HC | χ² | df | p |
| --- | --- | --- | --- | --- | --- | --- |
| Window Count  (mean ± SD) | 68.97 ± 25.14 | 69.43 ± 33.45 | 68.70 ± 27.96 | 1.26 | 2 | 0.53 |
| Sentence Count  (mean ± SD) | 70.97 ± 25.14 | 71.43 ± 33.45 | 70.71 ± 27.96 | 0.72 | 2 | 0.69 |
| Word count  (mean ± SD) | 1022.71 ± 362.08 | 995.1 ± 419.83 | 1074.54 ± 416.13 | 0.72 | 2 | 0.69 |

* *Bonferroni Correction,* Level of significance a=0.05

**S4. Language model**

The model uses a WordPiece tokenizer with a shared vocabulary of 30,522 tokens, resulting in embedding matrices of 1,024 dimensions, capturing bidirectional contextual information in text. While individual dimensions do not correspond to interpretable semantic features, meaningful representations arise from the joint geometry of the embedding vector.

The text was first tokenized into sentences and then partitioned into overlapping sliding windows of three consecutive sentences. Each window was encoded using the pre-trained Bert-large-german-cased model and fixed-length contextual embeddings, named window embeddings. These were extracted from the model’s hidden representations, specifically of the last layer, for subsequent quantitative analysis. The last layer of the pretrained model encodes high-level semantic information and discourse-level integration in addition to acoustic and syntactic information [17, 18].

In **Figure S1**, a conceptual representation of the $\lambda$ and LLE is presented, while **Figure S2** provides an example from one participant.

**Figure S1:** Lyapunov-inspired measure per speech window reflects embedding divergence across dimensions.
**(A)** Conceptual illustration of embedding trajectories within a speech window. Successive embedding states form a contextual representation in a high-dimensional semantic space, with local neighbourhoods tracking the evolution of nearby points. **(B)** Lyapunov-inspired measure (λ) computed per dimension indices (equation 6 above) for each speech window. Positive values (λ > 0, green) indicate local expansion, reflecting sensitivity to small perturbations, while negative values (λ < 0, red) indicate local contraction, reflecting stabilizing dynamics. The distribution of λ across dimensions reveals anisotropic dynamical behaviour in the embedding space, with different dimensions exhibiting distinct stability properties.

|  |  |
| --- | --- |

**Figure S2:** Representative λ and LLE values across windows from one subject.

**S5. Elastic net regression model**

Each subject was held out once for unbiased performance estimation in the outer cycle. In the inner loop, for each outer training set, we tuned hyperparameters using 5-fold CV inside an elastic-net regression model. This inner CV was performed only on the training data, not the held-out subject. Hyperparameters were selected within each outer fold, and the held-out subject was predicted using the model trained with those selected hyperparameters. The optimal elastic-net mixing parameter (α) was selected via nested five-fold cross-validation by minimizing the mean squared error (MSE); model performance was quantified using RMSE and coefficient of determination (R²=$1-\text{SSE}/\text{SST}$).

To evaluate the stability of these RMSE estimates, bootstrap resampling (1,000 iterations of subject and rerunning the analysis) was performed, resulting in 95% confidence intervals. In addition, we compared these permutation values with the real ones, and the false discovery rate (FDR_corrected p_values) was calculated.

**Table S3:** Results from the elastic-net regression analysis predicting clinical scores from transitions states of signs of λ and LLE of each window.

| **Clinical scores** | **R^2^** | **Permuted_FDR *p<0.01** | **RMSE CI** |
| --- | --- | --- | --- |
| SANS_Negative_FTD | 0.08 | <0.00* | 4.53 [3.56-5.42] |
| SANS_ Affective flattening | 0.09 | <0.00* | 4.56 [3.58-5.45] |
| SANS_Alogia | 0.01 | <0.00* | 8.13 [5.80-10.44] |
| SANS_Apathy | 0.04 | <0.00* | 2.40 [1.93-2.93] |
| SANS_Anhedonia | 0.05 | <0.00* | 3.91 [2.78-5.15] |
| SANS_Attention_Deficit | 0.04 | <0.00* | 12.37[10.9214.45] |
| SANS_withoutAttention_Deficit | 0.08 | <0.00* | 8.32 [6.70-9.88] |
| SAPS_Positive_FTD | 0.04 | <0.00* | 2.39 [1.91-2.91] |
| SAPS_Hallucinations | 0.00 | <0.00* | 5.78 [4.89-7.44] |
| SAPS_Delusions | 0.01 | <0.00* | 3.18 [5.49-6.54] |
| SAPS_BizarreBehavior | 0.00 | <0.00* | 1.40 [1.03-1.91] |
| * *Bonferroni Correction, Level of significance a=0.05* | | |  |

**Table S4:** Results of non-parametric ANOVA (Kruskal-Wallis) test between the two clusters using the sign of λ in the first window.

|  | **χ²** | **df** | **p** |
| --- | --- | --- | --- |
| SANS_Negative FTD | 3.96 | 1 | **0.047*** |
| SANS_ Affective flattening | 1.70 | 1 | 0.192 |
| SANS_Alogia | 2.09 | 1 | 0.148 |
| SANS_Apathy | 3.66 | 1 | 0.056 |
| SANS_Anhedonia | 4.19 | 1 | **0.041*** |
| SANS_Attention_Deficit | 4.88 | 1 | **0.027*** |
| SANS_withoutAttention_Deficit | 8.88 | 1 | **0.003*** |
| SAPS_Positive FTD | 0.73 | 1 | 0.394 |
| SAPS_Hallucinations | 2.20 | 1 | 0.138 |
| SAPS_Delusions | 0.00 | 1 | 1.000 |
| SAPS_BizarreBehavior | 2.42 | 1 | 0.120 |

* *Bonferroni Correction, Level of significance a=0.05*

**Table S5:** Results of the non-parametric ANOVA test between four groups formed by possible combinations of positive and negative lambda and LLE values.

|  | **χ²** | | **df** | | | **p** |  |
| --- | --- | --- | --- | --- | --- | --- | --- |
| SANS_Negative_FTD | | 6.86 | | 3 | 0.076 | | |
| SANS_ Affective flattening | | 7.32 | | 3 | 0.062 | | |
| SANS_Alogia | | 4.03 | | 3 | 0.258 | | |
| SANS_Apathy | | 4.04 | | 3 | 0.257 | | |
| SANS_Anhedonia | | 5.96 | | 3 | 0.113 | | |
| SANS_Attention_Deficit | | 7.02 | | 3 | 0.071 | | |
| SANS_withoutAttention_Deficit | | 11.03 | | 3 | **0.012*** | | |
| SAPS_Positive_FTD | | 1.20 | | 3 | 0.753 | | |
| SAPS_Hallucinations | | 3.12 | | 3 | 0.373 | | |
| SAPS_Delusions | | 1.51 | | 3 | 0.681 | | |
| SAPS_BizarreBehavior | | 5.84 | | 3 | 0.120 | | |

* *Bonferroni Correction, Level of significance a=0.05*
